# Exposure to inflammatory cytokines selectively limits GM-CSF production by induced T regulatory cells

**DOI:** 10.1002/eji.201444687

**Published:** 2014-10-01

**Authors:** Ben C Reynolds, Darryl G Turner, Rhoanne C McPherson, Catriona T Prendergast, Richard G Phelps, Neil A Turner, Richard A O'Connor, Stephen M Anderton

**Affiliations:** 1 MRC Centre for Inflammation Research, University of EdinburghEdinburgh, UK; 2 Centre for Immunity, Infection and Evolution, University of EdinburghEdinburgh, UK

**Keywords:** Autoimmunity, Cytokines, Immunotherapy, Regulatory T cells, Treg cells

## Abstract

Interest in manipulating the immunosuppressive powers of Foxp3-expressing T regulatory cells as an immunotherapy has been tempered by their reported ability to produce proinflammatory cytokines when manipulated in vitro, or in vivo. Understanding processes that can limit this potentially deleterious effect of Treg cells in a therapeutic setting is therefore important. Here, we have studied this using induced (i) Treg cells in which de novo Foxp3 expression is driven by TCR-stimulation in vitro in the presence of TGF-β. We show that iTreg cells can produce significant amounts of three proinflammatory cytokines (IFN-γ, GM-CSF and TNF-α) upon secondary TCR stimulation. GM-CSF is a critical T-cell derived cytokine for the induction of EAE in mice. Despite their apparent capacity to produce GM-CSF, myelin autoantigen-responsive iTreg cells were unable to provoke EAE. Instead, they maintained strong suppressive function in vivo, preventing EAE induction by their CD4^+^Foxp3^−^ counterparts. We identified that although iTreg cells maintained the ability to produce IFN-γ and TNF-α in vivo, their ability to produce GM-CSF was selectively degraded upon antigen stimulation under inflammatory conditions. Furthermore, we show that IL-6 and IL-27 individually, or IL-2 and TGF-β in combination, can mediate the selective loss of GM-CSF production by iTreg cells.

## Introduction

The effective therapeutic use of Foxp3^+^ Treg cell populations remains an important goal in the search for new treatments for autoimmune and allergic conditions, as well as for imposing tolerance toward transplanted organs. Both natural (n)Treg-cell and induced (i)Treg-cell populations have been shown to provide potent protection in mouse models when transferred before or after the onset of pathology 1–4. However, both of these Foxp3^+^ populations have also been shown to be able to “trans-differentiate” in vitro, producing proinflammatory cytokines following TCR stimulation in the presence of cytokines that drive Th1 or Th17 differentiation 5–7. Notably, subsets of human Treg cells can also display a capacity for IFN-γ 8,9 and IL-17 production 10–12 and experimental evidence suggests that the trans-differentiation of Treg cells might also occur in vivo 13,14. However, the majority of studies report beneficial results from transfer of Treg cell populations, indicating that in vivo processes can limit the risk of these cells having detrimental proinflammatory effects 15. Identifying these processes that might stabilize Treg cell suppressive function would therefore improve the chances of successful translation to the clinic.

We have previously described that TGF-β-induced iTreg cells do not produce IL-17 under Th17 conditions, because of their intrinsic production of small quantities of IFN-γ during their generation in vitro 16. While this prevents the production of one proinflammatory cytokine (IL-17) the other consequence is that iTreg cells are able to express T-bet and retain the ability to produce IFN-γ under secondary TCR stimulation, and this is greatly enhanced if that stimulation occurs in the presence of IL-12 16. In that previous study, we asked whether IL-12-conditioning would produce T-bet^+^, IFN-γ-producing “ex-iTreg” cells that could drive autoimmune pathology. We found that myelin-responsive ex-iTreg cells had a very poor ability to induce autoimmune CNS inflammation (EAE) when transferred, compared to T-bet^+^, IFN-γ-producing bona fide T effector (Teff) cells. That inability of ex-iTreg cells to drive profound pathology might have reflected the fact that IFN-γ is not a required cytokine for EAE pathology 17,18.

Here, we sought to understand whether iTreg cells could produce other proinflammatory cytokines and, if so, to identify what might limit such a response. We show that iTreg cells can also produce TNF-α and GM-CSF upon secondary stimulation in vitro in the absence of any exogenous cytokines. This did not impair their ability to suppress the activation of naïve T cells, either in vitro or in vivo. In contrast, upon receipt of TCR stimulation under inflammatory conditions in vivo (immunization with peptide in complete Freund's adjuvant, CFA), iTreg maintained their ability to produce both IFN-γ and TNF-α, but GM-CSF production was impaired.

## Results

### iTreg cells can produce IFN-γ, GM-CSF, and TNF-α upon secondary TCR stimulation

**Figure 1:**
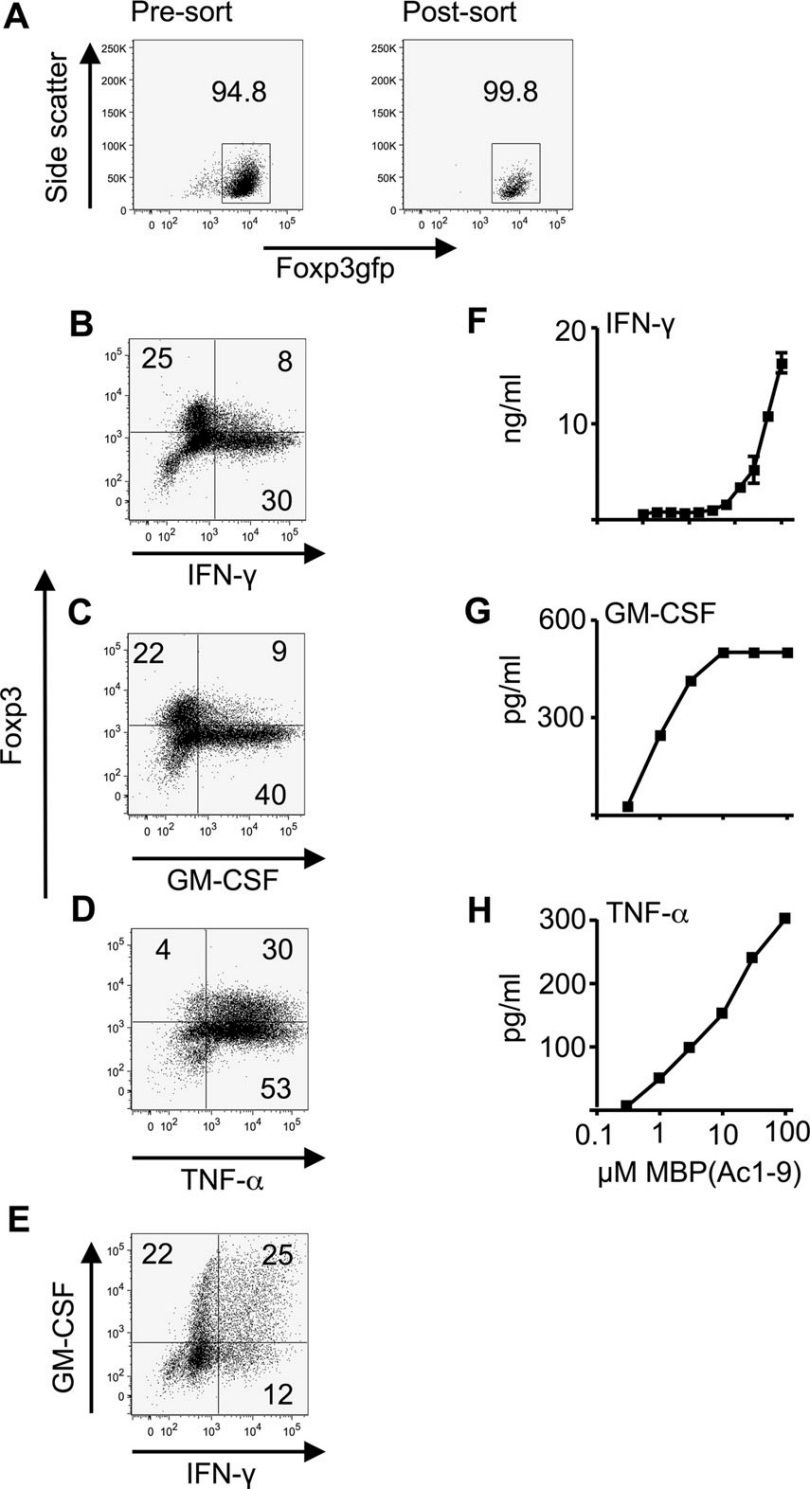
TGF-β-induced Treg cells produce pro-inflammatory cytokines IFN-γ, GM-CSF, and TNF-α. (A–E) iTreg cells were generated from CD4^+^Foxp3gfp^−^ cells (from Foxp3LuciDTR-4 mice) by 5-day culture as described in the *Materials and methods*. (A) Foxp3gfp expression pre and postsort on day 5 is shown. Numbers represent the percentage of Foxp3^+^ cells. (B–E) iTreg cells were then restimulated for 72 h with plate-bound anti-CD3 and anti-CD28 prior to intracellular staining for Foxp3 and cytokines and analysis by flow cytometry. Numbers on plots refer to percentage in each quadrant, rounded to the nearest integer. Data shown are from a single experiment representative of three experiments performed. (F–H) Tg4.Foxp3LuciDTR-4 iTreg cells (sorted to >98% Foxp3gfp^+^ purity) were restimulated for 48 h in triplicate with the indicated concentrations of MBP(Ac1–9) in the presence of splenic APCs. Supernatants were analyzed by ELISA for production of (F) IFN-γ, (G) GM-CSF, and (H) TNF-α. Data are shown as mean ± SEM of triplicates from one experiment representative of three experiments.

iTreg cells were generated by 5 day culture of naive (Foxp3-GFP^−^ CD4^+^) T cells with anti-CD3, anti-CD28, TGF-β, and IL-2. Highly pure iTreg cells were isolated by FACS-sorting based on Foxp3-GFP expression, (Fig. 1A) and were then restimulated with plate-bound anti-CD3 and anti-CD28 for 72 h. We used cytokine bead arrays to screen for the presence of inflammatory cytokines in supernatants from these secondary cultures. The majority of cytokines tested (IL-2, IL-4, IL-5, IL-6, IL-10, IL-13, IL-17, IL-21, and IL-22) were undetectable (data not shown). However, IFN-γ, TNF-α, and GM-CSF were detectable. Intracellular cytokine staining of restimulated iTreg cells demonstrated the loss of Foxp3 expression, as previously described 16, and confirmed the presence of cells staining positive for IFN-γ, GM-CSF, and TNF-α (Fig.1B–E). The majority of cells were TNF-α^+^ (Fig. 1D). Production of IFN-γ and GM-CSF was more restricted, and approximately 25% of cells were double positive for IFN-γ and GM-CSF (Fig. 1E). While IFN-γ and GM-CSF staining was seen chiefly in cells that had lost Foxp3 expression, TNF-α was also seen in a clear population that had retained Foxp3.

Loss of Foxp3 expression by iTreg cells is widely described, with the majority of cells here being Foxp3^−^ by the end of the 72 h secondary stimulation. We have previously studied this rigorously and have shown that contaminating Foxp3^−^ cells remaining after FACS sorting at the end of the primary iTreg-generating culture (here <1%) remain at the same frequency through to the end of the secondary culture 16. Therefore, these contaminating Foxp3^−^ cells could only account for ∼1% of cells in the culture at the time we assessed cytokine production, and could not be the source of the high frequencies of cytokine^+^ cells seen (Fig. 1B–E).

To be able to assay cytokine production in response to APCs presenting cognate antigen, we generated iTreg cells from Tg4.Foxp3.LuciDTR-4 mice, which express a transgenic TCR recognizing the Ac1–9 peptide of myelin basic protein (MBP). Supernatants of cultures in which these iTreg cells were restimulated using splenic APCs together with increasing concentrations of the MBP peptide demonstrated dose-dependent cytokine production. Of interest, TNF-α, and particularly GM-CSF production were evident at lower levels of TCR stimulation than were required for IFN-γ production (Fig. 1F–H).

### iTreg cells produce IFN-γ, GM-CSF, and TNF-α during their primary generation

**Figure 2:**
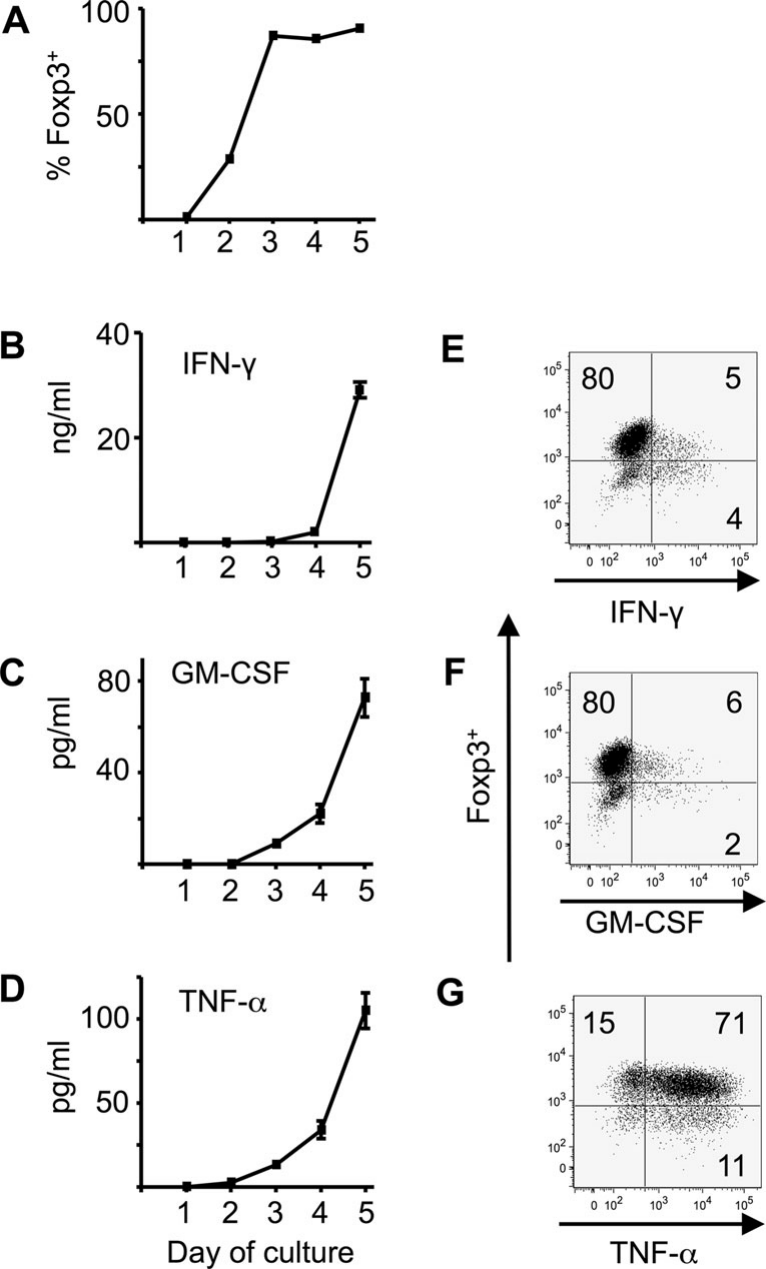
Production of IFN-γ, GM-CSF, and TNF-α occurs during primary iTreg-cell generation. Naive CD4^+^Foxp3gfp^−^ cells were cultured in triplicate for 5 days in iTreg-cell conditions (IL-2 and TGF-β with plate-bound anti-CD3 and anti-CD28). Cells and supernatants were sampled daily. (A) The percentage of cells expressing Foxp3 as determined by intracellular Foxp3 staining and flow cytometry is shown. (B–D) Supernatants were analyzed by ELISA for the presence of (B) IFN-γ, (C) GM-CSF, and (D) TNF-α. Data are shown as mean ± SEM of triplicates from one experiment representative of three experiments. (E–G) Flow cytometry at the end of iTreg-cell culture (day 5) showing intracellular staining for Foxp3 and (E) IFN-γ, (F) GM-CSF, and (G) TNF-α. Numbers on plots refer to percentage in each quadrant rounded to the nearest integer.

Cytokine production by iTreg cells was investigated further during the initial Foxp3-induction culture. Of note, Foxp3-gfp expression consistently increased to over 90% within 72 h (Fig. 2A). At that time, cytokine production was low or undetectable, but rose markedly in cultures sampled at days 4 and 5 (Fig. 2B–D). This argues against the possibility that the sole source of IFN-γ, GM-CSF, and TNF-α was cells that had not gained Foxp3 expression. This was further shown by clear populations of cytokine^+^ Foxp3^+^ cells at the end of the 5-day culture (Fig. 2E–F). This was particularly the case for TNF-α (Fig. 2F).

### Blockade of IFN-γ, GM-CSF, or TNF-α does not alter iTreg-cell suppressive function in vitro

**Figure 3:**
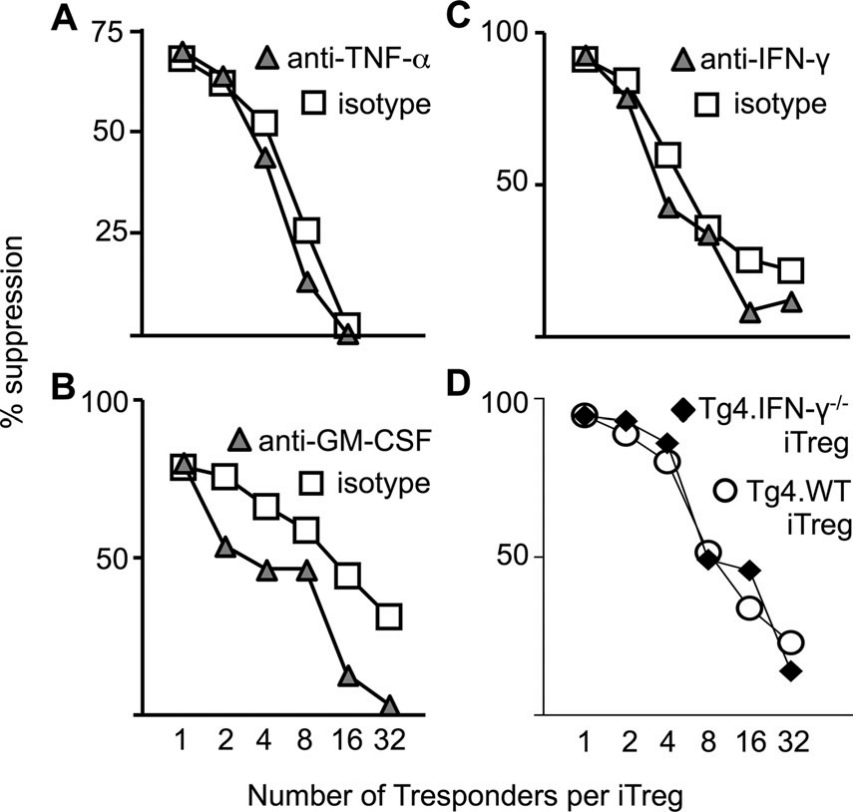
Production of IFN-γ, GM-CSF, and TNF-α is nonessential for iTreg-cell suppressive capacity. Varied numbers of iTreg cells were cocultured with a fixed number of naïve T responder cells as described in the *Materials and methods*. Proliferation was quantified by ^3 ^H-thymidine incorporation after 96 h culture. Percent suppression was calculated, based on the means of triplicate cultures, following the addition, at the onset of co-culture, of (A) anti-TNF-α, (B) anti-GM-CSF, and (C) anti-IFN-γ. Filled triangles: anti-cytokine, open squares: isotype control antibodies. (D) The suppressive capacity of iTreg cells generated from Tg4.IFN-γ^–/–^ mice were compared with that of iTreg cells generated from Tg4.CD90.1 (Tg4.WT) mice in suppression assays using naïve CD4^+^ Tg4 cells stimulated with MBP Ac1–9 and splenic APCs. Data are from one experiment representative of two experiments.

Thus far we had demonstrated that iTreg cells generated using a well characterized and widely used method would produce three proinflammatory cytokines upon secondary stimulation. We asked whether this would either diminish, or enhance, the strength of iTreg cell function using in vitro assays for suppression of naïve T cell activation. Although production of all three cytokines was again evident (data not shown), there was no apparent influence on the suppressive function of iTreg cells upon the proliferative response of naive T responder cells, stimulated by peptide-bearing APCs. Antibody neutralization of individual cytokines did not boost, or reduce, the observed suppression (Fig. 3A–C). Furthermore, IFN-γ-deficient iTreg did not have enhanced, or reduced, suppressive activity (Fig. 3D).

### Despite proinflammatory cytokine production, iTreg cells maintain suppressive activity in vivo

**Figure 4:**
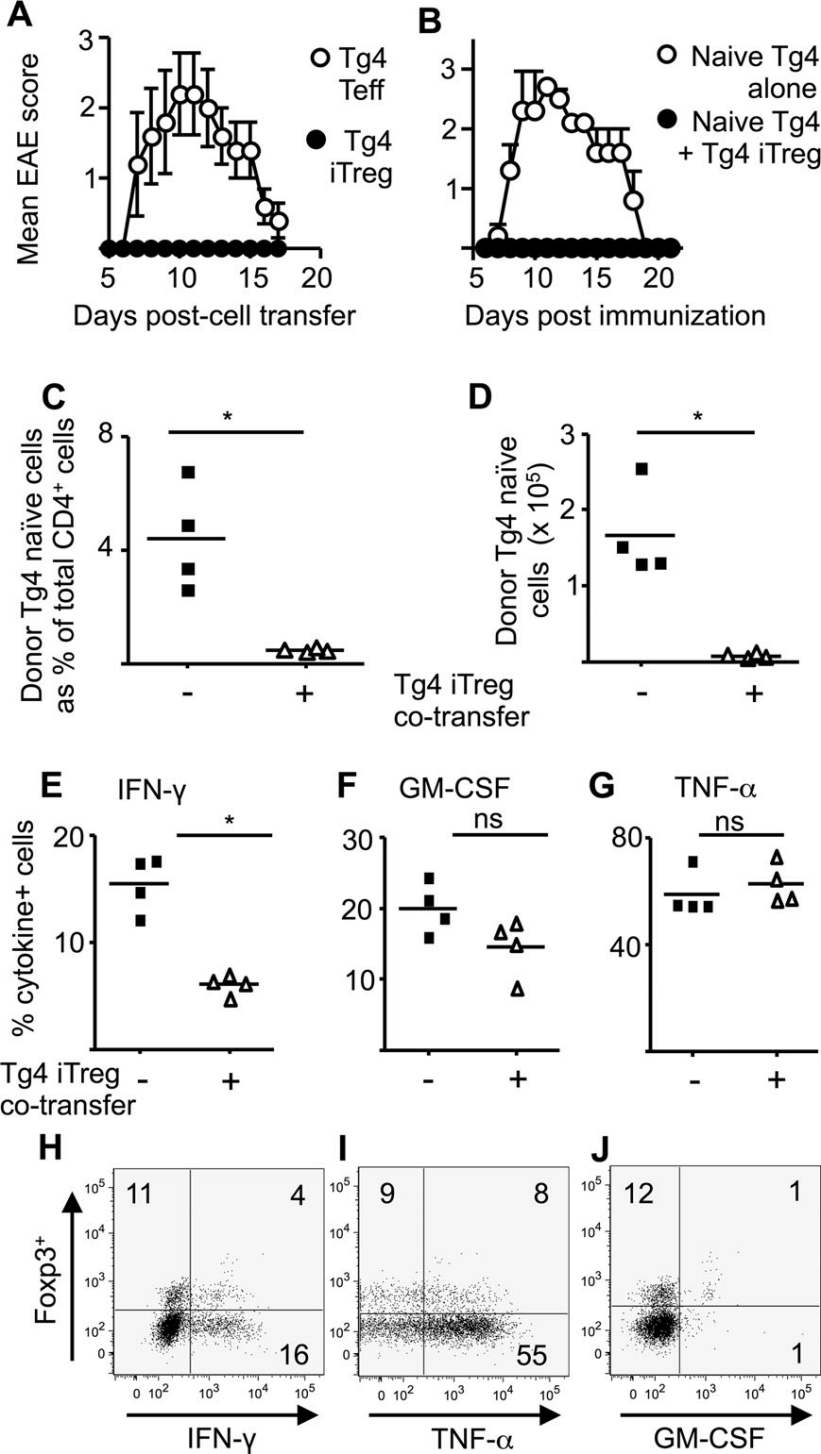
Autoantigen-responsive iTreg cells are suppressive rather than pathogenic and selectively lose the ability to produce GM-CSF in response to antigen in an inflammatory context. (A) Clinical course of passive EAE in C57BL/6 x B10.PL mice (five per group) that received either CD4^+^ effector cells (4 × 10^6^/mouse) or iTreg cells (6 × 10^6^/mouse) generated from naïve Tg4.Foxp3LuciDTR-4 T cells was monitored daily. (B) Clinical course of active EAE in C57BL/6 x B10.PL mice (five per group) that received 1 × 10^6^ naïve CD4^+^ Tg4 cells, either alone, or together with an equal number of Tg4 × Foxp3LuciDTR-4 iTreg cells, one day before immunization with MBP peptide, was monitored daily. Data are shown as mean ± SEM of the indicated number of mice from single experiments representative of two performed. (C–G) C57BL/6 × B10.PL mice (four per group) received 2 × 10^6^ naïve CD4^+^ Tg4.CD90.1 cells, either alone (–) or together with an equal number of Tg4.Foxp3LuciDTR-4 iTreg cells (+), one day before immunization with MBP peptide. After 7 days, draining lymph nodes were analyzed by flow cytometry for (C and D) the presence of the transferred naïve CD4^+^ Tg4.CD90.1 cells and (E–G) the ability of those cells to produce (E) IFN-γ, (F) GM-CSF, and (G) TNF-α after overnight culture of lymph node cells with the MBP peptide. Each data point represents an individual mouse and data shown are from single experiments representative of three performed. *p < 0.05 as determined by Mann–Whitney *U* test; ns = not significant. (H–J) B10.PL mice received 2 × 10^6^ Tg4 × Foxp3LuciDTR-4 iTreg cells alone one day before immunization with the MBP peptide as above. After 7 days, spleens were harvested and cultured and stained for cytokine production as above. Plots are gated on CD45.1^+^ donor iTreg cells (for gating strategy, see Supporting Information Fig. 2) showing expression of Foxp3 and production of (H) IFN-γ, (I) TNF-α, and (J) GM-CSF. Numbers on plots refer to percentage in each quadrant, rounded to the nearest integer. Data shown are from a single experiment representative of three performed.

iTreg cells can therefore produce GM-CSF, IFN-γ, and TNF-α, as well as showing expression of T-bet 16. This phenotype is commonly seen in myelin-responsive CD4^+^ T cells that can induce passive EAE upon adoptive transfer 19–21. We have previously reported only very poor pathogenic activity with “ex-iTreg” cells (which in that case had received a second stimulation in the presence of IL-12 to further boost T-bet expression and IFN-γ production) 16. However, we reasoned that those cells might have had reduced GM-CSF production, due to the inhibitory effect of IL-12 20. Given that T cell production of GM-CSF (and not IFN-γ) is now believed to be essential for EAE induction 17,18,20,22,23, we revisited this issue, by testing the pathogenic activity of GM-CSF-producing primary iTreg cells (not exposed to IL-12). Using MBP-responsive iTreg cells generated from Tg4.Foxp3LuciDTR-4 mice, we found that transfer of these cells did not provoke clinical signs of EAE (Fig. 4A). Moreover, we confirmed the maintained suppressive activity of these autoreactive iTreg cells, because their cotransfer alongside naive Tg4 T responder cells into C57BL/6 × B10.PL mice prevented EAE upon subsequent immunization with the MBP peptide in CFA (Fig. 4B).

The donor T responder cell population was distinguishable by its unique expression of CD90.1, allowing assessment of the effects of iTreg cells upon their naive counterparts (Fig. 4C–G). The presence of iTreg cells limited the numbers and frequencies of T responders found in the draining lymph nodes sampled 7 days after immunization (Fig. 4C, D). Interestingly, comparison of cytokine production by the T responder population revealed that it was only the frequencies of IFN-γ^+^ (not TNF-α^+^ or GM-CSF^+^) cells that were diminished in this population when iTreg cells were also administered (Fig. 4E–G). However, the significantly lower numbers of T responders (Fig. 4D) meant that total numbers of all cytokine^+^ T responders were lower when iTreg cells were present in the priming lymph node. We therefore concluded that the suppressive effects of iTreg cells upon T responders can proceed in vivo despite the ability of iTreg cells to produce IFN-γ, GM-CSF, and TNF-α.

### iTreg cells do not produce GM-CSF when stimulated under inflammatory conditions in vivo

To justify the above conclusion, we performed experiments to confirm that iTreg cells maintained their ability to produce cytokines in the in vivo inflammatory setting used (immunization with cognate peptide in the presence of CFA). Tg4.Foxp3LuciDTR-4 iTreg cells were transferred alone, with immunization the following day. Donor iTreg cells (identified by expression of CD45.1) sampled 7 days later had largely lost Foxp3 expression, but maintained the ability to produce IFN-γ and TNF-α (Fig. 4H, I). In contrast, their ability to produce GM-CSF was markedly impaired (Fig. 4J). Analysis of host CD4^+^ cells confirmed the presence of Foxp3^−^ GM-CSF^+^ cells, demonstrating that this finding was not due to technical failure of anti-GM-CSF staining.

### iTreg cells remain suppressive following secondary stimulation, despite loss of Foxp3 expression

The data above indicated that the iTreg-cell population was suppressive following in vivo immunization (Fig. 4B) despite largely losing Foxp3 expression (Fig. 4H–J). We sought to test whether this was due to retained suppressive activity in cells that had lost Foxp3, or to overriding suppression provided by a minor population that had maintained Foxp3. iTreg cells were generated and subjected to secondary TCR stimulation in vitro. As seen above (Fig. 1), this drove the loss of Foxp3-GFP expression in a proportion of cells, allowing us to sort into GFP^+^ and GFP^−^ populations (Supporting Information Fig. 1). These were then tested in in vitro suppression assays. Inhibition of the proliferation of responder cells was equivalent regardless of the GFP status of the iTreg cells used (Supporting Information Fig. 1C). We conclude that iTreg cells can maintain suppressive activity once Foxp3 is lost, at least for the duration of an in vitro suppression assay.

### Exposure to cytokines inhibits the ability of iTreg cells to produce GM-CSF

**Figure 5:**
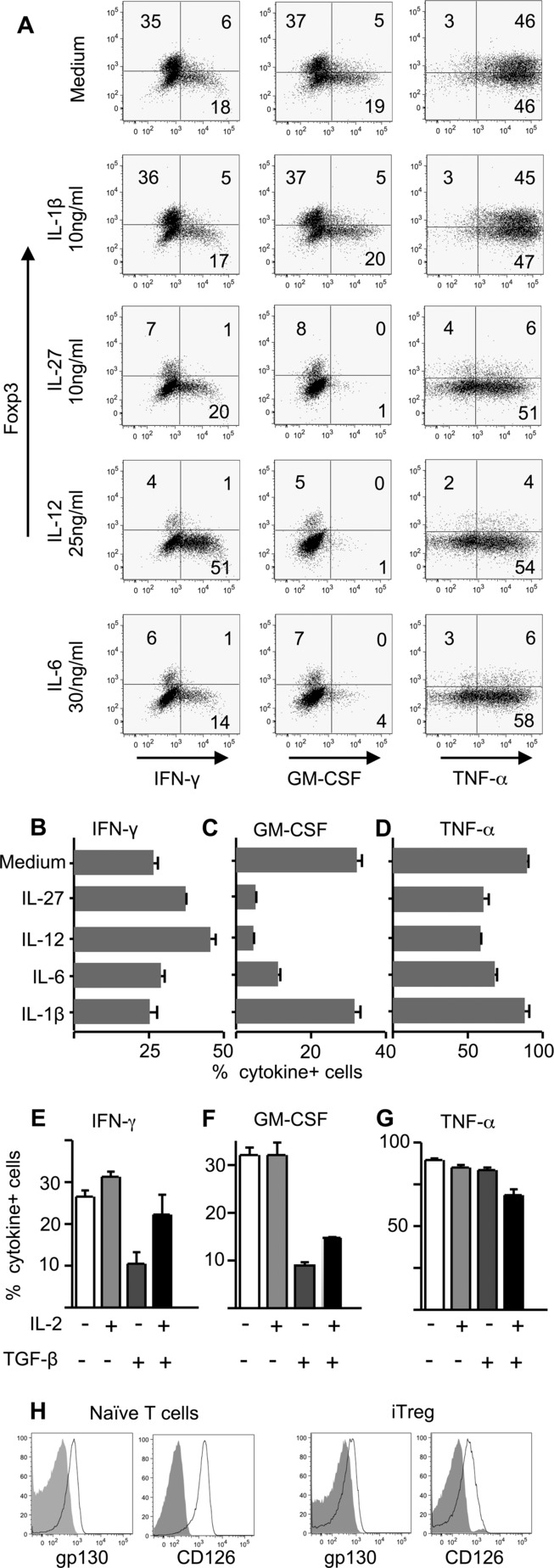
Proinflammatory cytokines can selectively impair the production of GM-CSF by iTreg cells. (A–D) Sorted (>99% Foxp3gfp^+^) iTreg cells were restimulated with plate-bound anti-CD3 and anti-CD28 (both 2 μg/mL) with the addition of IL-12 (25 ng/mL), IL-27 (10 ng/mL), IL-6 (30 ng/mL), or IL-1β (10 ng/mL) for 72 h, with brefeldin A, PMA, and ionomycin for the final 4 h of culture. Cytokine production was then assessed by intracellular staining. (A) Representative flow cytometry plots of cytokine production by iTreg cells gated on live CD4^+^ cells (for gating strategy, see Supporting Information Fig. 3). Numbers on plots refer to percentage in each quadrant, rounded to the nearest integer. (B–D) Summary data for the proportion of restimulated iTreg cells producing (B) IFN-γ, (C) GM-CSF, and (D) TNF-α are shown as mean + SEM of triplicates. (E–G) iTreg cells were restimulated in the presence or absence of additional IL-2 (100 U/mL) and/or TGF-β (5 ng/mL), then analyzed as above. Summary data show the proportion of iTreg cells producing (E) IFN-γ, (F) GM-CSF, and (G) TNF-α are shown as mean ± SEM of triplicates. All data are from one experiment representative of three independent experiments. (H) iTreg cells were stained at the end of the 5-day Foxp3-induction culture for the expression of gp130 and CD126 (open histograms). Filled histograms show isotype control staining. Naïve T cells were also stained.

The results in Fig. 4H–J suggested that component(s) of the in vivo inflammatory milieu were capable of selectively degrading the ability of iTreg cells to produce GM-CSF while maintaining IFN-γ and TNF-α production. To understand whether inflammatory cytokine(s) might be responsible for this, we returned to the in vitro restimulation of iTreg cells either under “neutral” conditions, or in the presence of additional cytokines (Fig. 5).

Inclusion of IL-1β did not modify cytokine production by iTreg cells (Fig. 5A–D). Both IL-12 and IL-27 are known to inhibit GM-CSF production, but to promote/maintain IFN-γ production, by CD4^+^ T cells 20,24. We found that addition of either of these cytokines could specifically suppress GM-CSF, but not IFN-γ or TNF-α, recapitulating the ex vivo response profile of iTreg cells following immunization (Fig. 5A–D). Increased frequencies of IFN-γ^+^ cells seen in iTreg cells restimulated in the presence of IL-12 (Fig. 5A and B) were consistent with our previous observations 16. Inclusion of IL-6 in iTreg-cell restimulation cultures also reduced the frequencies of GM-CSF^+^ cells (Fig. 5A and C).

Inflamed lymph nodes contain other cytokines relevant to Treg cells, in particular IL-2 and TGF-β. Inclusion of IL-2 alone did not alter the frequencies of cells staining for either of the three cytokines of interest (Fig. 5E–G). In contrast, the known suppressive effects of TGF-β on IFN-γ production were evident in these iTreg cell cultures (Fig. 5E). Inclusion of TGF-β also lowered the frequencies of GM-CSF^+^ cells (Fig. 5F), but had no effect on TNF-α^+^ frequencies (Fig. 5G). Inclusion of IL-2 and TGF-β together restored IFN-γ (Fig. 5E), but did not restore GM-CSF expression.

Consistent with their sensitivity to IL-6 (Fig. 5A and C), iTreg cells expressed both chains of the IL-6 receptor (gp130 and CD126), albeit to lower levels than found on naive CD4^+^ T cells (Fig. 5H).

### Proinflammatory cytokines differentially affect cytokine production by Th0, Th1, and iTreg cells

**Figure 6:**
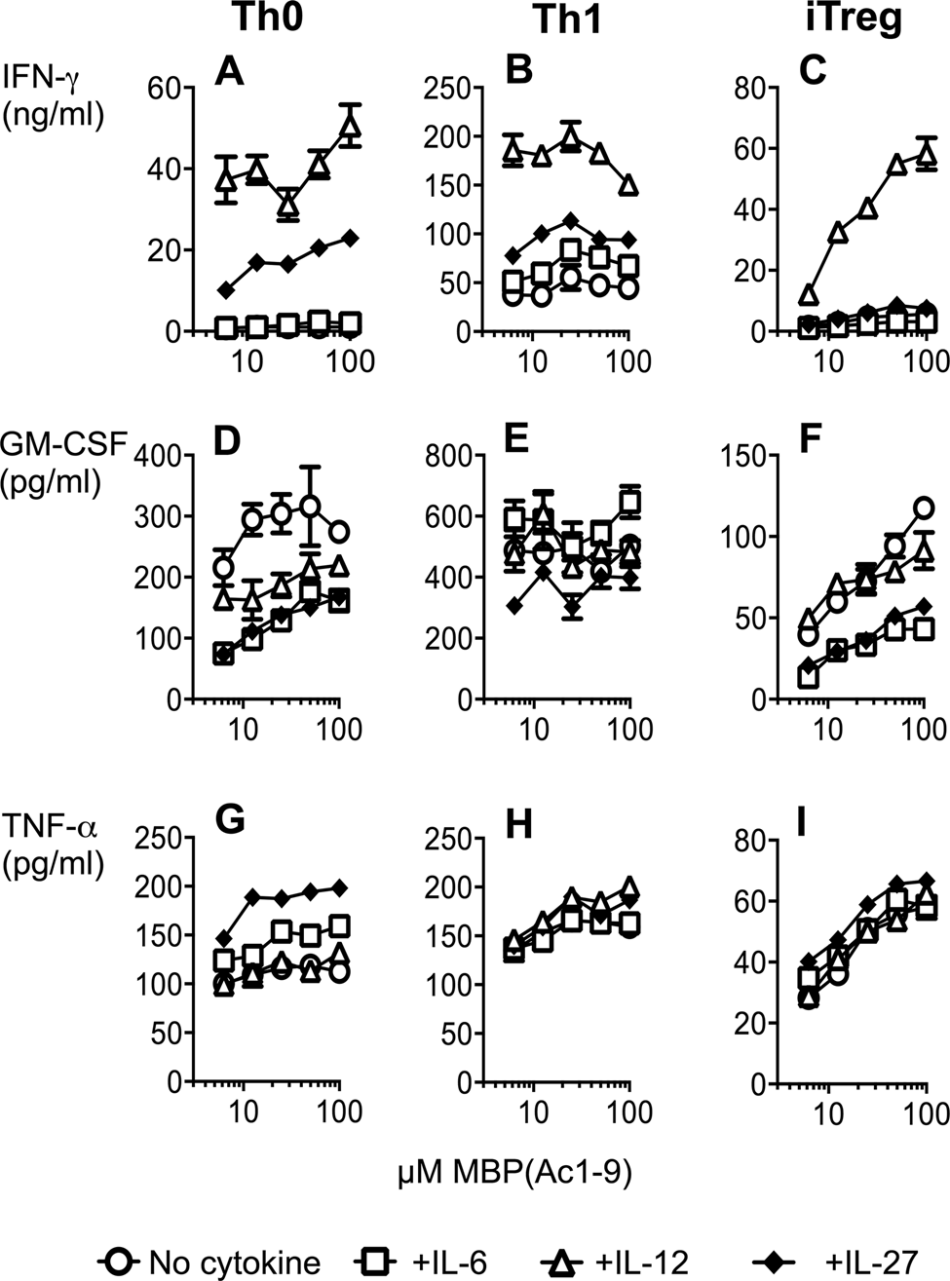
Differential influence of IL-6, IL-12, and IL-27 upon cytokine production by Th0, Th1, and iTreg cells. Th0, Th1, and iTreg cells were generated from naive CD4^+^ Tg4.Foxp3LuciDTR-4 cells. Prior to restimulation, cells were FACS-sorted as GFP^+^ (for iTreg cells) or GFP^−^ (for Th0 and Th1 cells). Cells were then restimulated for 48 h in triplicate with B10.PL splenic APCs and a dose response of MBP(Ac1–9) peptide with or without the addition of IL-6 (30 ng/mL), IL-12 (25 ng/mL), or IL-27 (50 ng/mL). Supernatants were analyzed by ELISA for production of the indicated cytokines. Data are shown as mean ± SEM of triplicates from one experiment representative of two experiments.

We addressed whether the inhibitory effects of IL-6, IL-12, and IL-27 upon cytokine production were a unique feature of iTreg cells, or were common to other CD4^+^ T cell populations capable of producing IFN-γ, GM-CSF, and TNF-α (Fig. 6). For this comparison, iTreg, Th0, and Th1 cells were generated from naïve Tg4.Foxp3LuciDTR-4 cells and restimulated with splenic APCs and a dose response of MBP peptide. In the absence of exogenous cytokines, iTreg cells produced lower levels of GM-CSF and TNF-α than their Th0 and Th1 counterparts. Levels of IFN-γ were similar between iTreg cells and Th0 cells and, as would be expected, these were lower than those detected from Th1 cells (Fig. 6A–C). Addition of either IL-6 or IL-27 inhibited iTreg-cell production of GM-CSF (Fig. 6F), mirroring their effects seen with APC-free TCR stimulation (in Fig. 5). However, inhibition of GM-CSF was not seen with IL-12. GM-CSF production by iTreg cells and Th0 cells was affected in a similar way by exogenous cytokines (Fig. 6D and F), whereas no clear inhibition of Th1-cell production of GM-CSF was evident (Fig. 6E). The only cytokine to clearly elevate IFN-γ production by iTreg cells was IL-12; IL-27 could not achieve this (Fig. 6C). We conclude that, while there is some overlap in how IL-6, IL-12, and IL-27 influence cytokine production by Th0, Th1, and iTreg cells, each cell-type shows a unique response profile to these cytokines.

## Discussion

Despite having potent suppressive activity in vitro and in vivo, Treg cells possess the ability to produce significant amounts of proinflammatory cytokines when stimulated in a range of inflammatory milieu 15. These concerns are compounded by the consistent observations that, in in vivo models the most efficacious Treg cells are those that recognize an antigen that is relevant to the disease being studied, presumably because these Treg cells have an improved capacity to reach the organ in which their antigen is concentrated and/or to persist there 1,2. In the therapeutic setting, this organ would be inflamed and therefore the infiltrating Treg cells would encounter cytokines that might promote their trans-differentiation toward effector function. The therapeutic benefit of antigen-specific Treg cells would be greatly outweighed by the clinical risks if these cells became pathogenic, as further organ destruction would seem almost inevitable. Understanding this possible trans-differentiation of Treg cells thus assumes great importance in ensuring their translation to the therapeutic armamentarium.

Here, we extend our previous observation that iTreg cells can produce significant amounts of IFN-γ 16, to show that they also can produce two other potent inflammatory cytokines, GM-CSF, and TNF-α. The production of these three proinflammatory cytokines by iTreg cells is gained during their primary generation in the presence of TGF-β. Indeed, the concentration of all three cytokines appears to coincide with the expression of Foxp3 in primary iTreg-cell cultures. Thus, the production of these cytokines appears to be intrinsic to iTreg cells, rather than representing a loss of their suppressive phenotype. Neutralization of either cytokine did not impact on the ability of iTreg cells to suppress naïve T cells, at least within the systems investigated here. This raises the question of whether there is a functional (suppressive) purpose to the production of these cytokines by iTreg cells?

The intrinsic ability of iTreg cells to express T-bet and produce IFN-γ could be enhanced by exposure to IL-12 16,25. Despite this, in our previous study we found that MBP-responsive iTreg cells exposed to IL-12 could produce only very modest clinical signs of CNS inflammation (far weaker than those provoked by bona fide Teff cells expressing the same MBP-responsive TCR), even when infused in relatively high numbers. In fact, those IFN-γ-producing iTreg cells were able to suppress EAE driven by naïve MBP-responsive T cells following immunization 16. Similarly, microbiota-responsive IFN-γ^+^Foxp3^+^ iTreg cells failed to induce colitis upon transfer to RAG^−/−^ hosts, instead retaining suppressive function, protecting against disease transferred by Foxp3^−^ T cells 25. A protective role for IFN-γ and a contribution of this cytokine to suppressive function in vivo has been suggested in a skin graft tolerance model 26. There, mice tolerized to skin grafts by the infusion of alloantigen-responsive Treg cells rapidly rejected the grafts following treatment with anti-IFN-γ. Human IFN-γ^+^Foxp3^+^ Treg cells can suppress alloresponses in vitro and the presence of elevated levels of IFN-γ^+^Foxp3^+^ T cells in peripheral blood correlated positively with good long-term graft function following renal transplantation 27. Elevated frequencies of IFN-γ^+^ Treg cells have also been identified in human patients with diabetes mellitus and these were shown to have suppressive function in vitro 9. Notably, those IFN-γ^+^Foxp3^+^ Treg cells lacked Helios expression and showed incomplete demethylation of the Treg-specific demethylation region within the Foxp3 promoter, indicating that they were peripherally generated (p)Treg cells 9. The induced coexpression of Foxp3 and IFN-γ therefore represents an important element of naturally occuring immunoregulatory networks rather than an in vitro artifact. IFN-γ has a protective role in EAE 18, and Treg cells have a key suppressive function within the inflamed CNS 28,29. However, we have previously reported that Treg cells isolated from the inflamed CNS do not produce IFN-γ 30, indicating that this cytokine is not a critical component of their suppressive armory in that context. Indeed, others have reported that production of IFN-γ by the EAE-initiating T cells is important for determining EAE severity 31.

Treg cells have greater expression of TNF receptor 2 than other activated T cells, and this may be correlated with their suppressive function 32. A crucial role for TNF-α has been suggested in suppression by nTreg cells in a model of colitis in RAG^–/–^ mice 33. Treg-cell function has been reported to be enhanced in the presence of Teff cells, and this was partly mediated through Teff-cell production of TNF-α 34. Thus, iTreg cells may similarly boost their own suppressive function in an autocrine fashion, although here neutralization of TNF-α did not modify in vitro suppressive function.

The recent reemphasis on GM-CSF as a key cytokine produced by encephalitogenic T cells 20 gave a possible explanation for the relative failure of IFN-γ-producing iTreg cells to produce pathology 16 (IFN-γ is not required for EAE induction, but GM-CSF is). IL-12-conditioning of iTreg cells elevated their production of IFN-γ, but would diminish their ability to produce GM-CSF (as shown here). However, administration of high numbers of MBP-responsive iTreg cells, with a retained capability for GM-CSF production, could not deliver clinical signs of autoimmune CNS inflammation. Rather, they maintained profound suppressive function. It was notable that suppressive iTreg cells largely lost Foxp3 expression after transfer and immunization, analogous to the loss of Foxp3 upon secondary in vitro stimulation. In the in vitro scenario, iTreg cells that had lost Foxp3(GFP) were equally suppressive as their Foxp3(GFP)^+^ counterparts. The in vivo experimental system used was not amenable to verification of this, but these data indicate that suppressive function of iTreg cells can outlast their expression of Foxp3. Further detailed investigation will be needed to understand the basis and longevity of this suppressive function in the absence of sustained Foxp3-expression.

Our data indicate that, rather than gaining a proinflammatory function, iTreg cells lose this capacity (at least to some extent) when placed in an inflammatory environment. Following immunization with myelin autoantigen in CFA, the myelin-responsive naive T cells that go on to form encephalitogenic Teff cells rapidly gain the ability to produce inflammatory cytokines, including IFN-γ, TNF-α, and GM-CSF 35. When MBP-responsive iTreg cells were exposed to this in vivo inflammatory scenario, they maintained their capacity to produce IFN-γ and TNF-α, but their ability to produce GM-CSF was relatively degraded. In vitro experiments showed that this phenotype could be replicated by several, but not all, cytokines tested. Two of these, IL-1β and IL-2, when given alone, did not alter the frequencies of iTreg cells making IFN-γ, GM-CSF, or TNF-α. The documented reduction in GM-CSF production by Teff cells exposed to IL-12 or IL-27 24, could be replicated in iTreg cells. IFN-γ and TNF-α were not grossly altered by exposure IL-27, whereas IL-12 selectively elevated IFN-γ, as we reported previously 16. Exposure to IL-6 could also reduce GM-CSF production while leaving IFN-γ and TNF-α intact. TGF-β suppressed GM-CSF production, but also (as has been well documented for the control of Th1 responses 21,36,37) greatly reduced IFN-γ production (not a feature of iTreg cells exposed to in vivo stimulation, in the inflamed lymph node). However, while IL-2 alone had no effect on cytokine production by iTreg cells, it could counteract the TGF-β-mediated inhibition of IFN-γ, but not GM-CSF. Therefore, the altered cytokine profile displayed by iTreg cells placed in an inflamed lymph node could be replicated in vitro by exposure to IL-12 alone, IL-27 alone, IL-6 alone, or a combination of TGF-β plus IL-2. All of these cytokines would be present at elevated concentrations in an inflamed lymph node, and most likely an inflamed tissue. Furthermore, the selective inhibitory effect of IL-27 upon GM-CSF production (i.e. without boosting IFN-γ production) by iTreg cells was not replicated in Th0 or Th1 cells, which did show elevated IFN-γ production. IL-12 boosted IFN-γ production by iTreg cells, but only suppressed their production of GM-CSF when TCR stimulation was provided in the absence of APCs. This was unlikely to reflect a constitutive inhibition of GM-CSF by APC-derived IL-12, that could not be enhanced by exogenous IL-12, since Th0 production of GM-CSF was inhibited by IL-12 addition. The selective inhibition of cytokine production by Treg cells under different inflammatory milieu warrants further investigation.

The expression of cytokine receptors by T cells seems quite dynamic, varying with stage of (and time since) activation. For example, although IL-6 is entirely required for the induction of EAE 38,39, this is a transient requirement 40,41 and by the time T cells (both Teff and Treg cells) arrive in the CNS they are insensitive to IL-6, having downregulated expression of both gp130 and CD126 30. Considering this point, the inhibitory effect of IL-6 upon iTreg-cell production of GM-CSF was somewhat surprising, given a previous report describing enhanced loss of CD126 at 3 days following activation in primary iTreg-generating cultures 6. This difference is resolved by our observation that, at the time of secondary stimulation, our iTreg cells did show expression of gp130 and CD126. Whether this was a kinetic factor, i.e. the iTreg cells had lost but then regained these receptor chains during the 5 days of culture, remains to be defined. Clearly iTreg cells express receptors for IL-2 and TGF-β (both key to their generation) and must also express receptors for IL-12 and IL-27, when exposed to these cytokines in the cultures used here. We suggest that the dynamic expression of cytokine receptors (not just by T cells) during inflammation is a very important, but largely neglected area of study. Furthermore, given our observations, investigations into the stability of Treg cells should not use evidence of proinflammatory cytokine production as a surrogate for evidence of loss of suppressive function.

## Materials and methods

### Mice, antigens, and tissue culture medium

C57BL/6, B10.PL, C57BL/6xB10.PL, Foxp3GFP, Foxp3LuciDTR-4 42, Tg4.CD90.1, and Tg4.Foxp3LuciDTR-4.CD45.1 16 mice were bred under specific pathogen-free conditions. Tg4.CD45.1.IFN-γ^−/−^ were generated by crossing IFN-γ^–/–^ mice with Tg4.CD45.1 21 mice and backcrossing for ten generations. Experiments received University of Edinburgh ethical approval and were performed under UK. legislation (PPL 60/4116). The acetylated 1–9 peptide of MBP (ASQKRPSQR) and the Ac1–9(4Tyr) variant were synthesized by Cambridge Research Biomedicals (Cambridge, UK). Tissue culture medium was RPMI 1640 containing 10% heat-inactivated fetal calf serum (Sigma-Aldrich, Poole, UK.), 2 mM l-glutamine, 100 U/mL penicillin, 100 μg/mL streptomycin (all PAA Laboratories Ltd., Somerset, UK) and 50 μM 2-β-mercaptoethanol (Gibco).

### T-cell purification and iTreg-cell generation

CD4^+^ T cells were purified using magnetic cell sorting (autoMACS Pro, Miltenyi Biotec, Bergisch Gladbach, Germany) as per the manufacturer's instructions, with or without subsequent FACS-sorting. Naïve CD4^+^Foxp3^−^ T cells from Foxp3-reporter mice were isolated and purified (routinely to >99% CD4^+^Foxp3^−^) by FACS-sorting on a BD FACSARIA II (BD Biosciences, Franklin Lakes, NJ). Naïve CD4^+^ T cells from Tg4.CD45.1.IFN-γ^–/–^ mice (lacking a Foxp3-reporter) were purified using a naïve CD4^+^ T cell isolation kit (Miltenyi Biotec) as per the manufacturer's instructions. Foxp3 expression was induced by stimulation with plate-bound anti-CD3 + anti-CD28 (both at 2 μg/mL) for 5 days in the presence of 100 U/mL IL-2 and 5 ng/mL recombinant human TGF-β1 (R&D Systems, Minneapolis, MN). In some experiments, daily aliquots of culture supernatants were sampled for detection of cytokine production by ELISA. On day 5, Foxp3^+^ cells were FACS-sorted by Foxp3-reporter expression (routinely to >99% purity).

### iTreg-cell suppression assays

Suppression assays were performed by culturing naïve CD4^+^ T responder cells (2 × 10^4^/well) for 96 h with increasing numbers of iTreg in the presence of 1 × 10^5^ irradiated (30 Gy) APCs (red cell-lysed B10.PL or C57BL/6 × B10.PL splenocytes) and either 1 μg/mL anti-CD3 or 10 μM MBP Ac1–9 peptide. A 0.5 μCi ^3^H-thymidine (Amersham Biosciences, Amersham, UK.) was added for the final 16 h and incorporation measured by β-scintillation counting (Wallac, Turku, Finland). Where indicated, 10 μg/mL of the following antibodies were added individually at the onset of culture: rat anti-mouse GM-CSF (clone MP1–22E9, BD Pharmingen), rat anti-mouse TNF-α (clone G281–2626, BD Pharmingen), mouse anti-IFN-γ (clone XMG1.2, Bioxcell, West Lebanon, NH).

### FACS analysis of cytokine production

Surface staining was performed prior to processing for Foxp3 staining using Foxp3 fix/perm buffers according to the manufacturer's instructions (e-bioscience). Cells were then stained for intracellular cytokines and appropriate isotype controls. The following antibodies and isotype controls were used (all from eBioscience unless stated); anti-CD4-e450, anti-CD4-AF700 (BD Biosciences), anti-CD90.1-allophycocyanin, anti-CD90.1-FITC, anti-CD45.1-PerCPCy5.5, anti-gp130-allophycocyanin, anti-CD126-PE, anti-Foxp3-e450, anti-Foxp3-FITC, anti-Foxp3-allophycocyanin, anti-IFN-γ-FITC, anti- IFN-γ-allophycocyanin, anti-GM-CSF-PE (BD Biosciences), anti-TNF-e450, anti-CD25-PE, anti-CD25-PerCPCy5.5 (BioLegend UK), rat IgG1-(FITC/PE/allophycocyanin/e450/AF647), rat IgG2a-(PE/e450/allophycocyanin/FITC/PerCPCy5.5), rat-IgG2b-allophycocyanin, viability dye e780. Following acquisition (LSR Fortessa, II BD Biosciences), analysis was performed using FlowJo software (Treestar version 3.2.1.).

In some experiments cytokine production was determined by intracellular staining immediately after the 5-day iTreg-cell culture. Cells were incubated at 37°C in the presence of 50 ng/mL PMA, 1 μg/mL ionomycin (Sigma), and 3 μg/mL brefeldin A (eBioscience) for 4 h prior to staining.

In other experiments iTreg cells were restimulated by 72 h culture for with anti-CD3 and anti-CD28 (both 2 μg/mL). Where indicated, the following cytokines were added individually to triplicate wells: IL-12 25 ng/mL (R&D), IL-27 10 ng/mL (R&D), IL-6 30 ng/mL (Miltenyi), IL-23 30 ng/mL (R&D), IL-1β 10 ng/mL (R&D), IFN-γ 100 ng/mL (BD), TGF-β 10 ng/mL (R&D), IL-2 100 U/mL.

### Generation of Tg4 Th0 and Th1 cells

Th0 and Th1 populations were generated from naive CD4^+^ Tg4.Foxp3LuciDTR-4 cells (FACS-sorted CD4^+^CD62L^hi^CD25^−^Foxp3-GFP^−^) by 72 h stimulation with plate-bound anti-CD3 + anti-CD28. Th0 cultures were supplemented with 10 U/mL IL-2 and 10 μg/mL anti-IFN-γ. Th1 cultures were supplemented with 25 ng/mL IL-12, 25 ng/mL IL-18, and 10 U/mL IL-2 (at 48 h, a further 20 U/mL IL-2 was added). After 72 h, cells were replated with fresh culture medium containing 10 U/mL IL-2 for a further 48 h, at which time cells were FACS-sorted for GFP^−^ cells.

### Tg4 T cell responses to antigen

Tg4 iTreg, Th0, or Th1 cells (2 × 10^4^/well) were stimulated with increasing concentrations of the MBP (Ac1–9) peptide in the presence of irradiated (30 Gy) splenic APCs (1 × 10^5^/well). After 48 h supernatants were collected for analysis of cytokine production by ELISA.

For ex vivo analysis of Tg4 T-cell (Tresponder or iTreg) responses, lymphoid cell populations were cultured overnight with 10 μM MBP(Ac1–9) with the addition of brefeldin A for the final 4 h and processing for intracellular cytokine staining as above.

### In vivo immunization and EAE induction

Host C57BL/6×B10.PL mice received an i.v. infusion of 1–2 × 10^6^ naïve CD4^+^ Tg4.CD90.1 T responder cells, or Tg4.Foxp3LuciDTR-4.CD45.1 iTreg cells, either alone, or together at a 1:1 ratio. One day later, mice were immunized with 10 μg of MBP Ac1–9(4Tyr) emulsified in CFA (containing 50 μg heat-killed *Mycobacterium tuberculosis* H37Ra) (Sigma) at a final volume of 100 μL as described 16. For priming experiments, lymphoid organs were isolated 7 days after immunization. For EAE experiments host mice also received 200 ng pertussis toxin (Health Protection Agency, Porton Down, UK) i.p. on the day of immunization and 2 days later. For passive transfer of EAE, host C57BL/6 × B10.PL mice received an i.v. injection of 4 × 10^6^ CD4^+^ Tg4 effector T cells generated by primary in vitro stimulation as described 21, or 6 × 10^6^ Tg4.Foxp3LuciDTR-4.CD45.1 iTreg cells. Mice also received pertussis toxin as above. Clinical signs of EAE were assessed as described 21.

## Abbreviations

CFA: complete Freund's adjuvant
iTreg cell: induced Treg cell
MBP: myelin basic protein

## References

[1] Stephens LA, Malpass KH, Anderton SM (2009). Curing CNS autoimmune disease with myelin-reactive Foxp3+ Treg. Eur. J. Immunol.

[2] Tang Q, Henriksen KJ, Bi M, Finger EB, Szot G, Ye J, Masteller EL (2004). In vitro-expanded antigen-specific regulatory T cells suppress autoimmune diabetes. J. Exp. Med.

[3] DiPaolo RJ, Brinster C, Davidson TS, Andersson J, Glass D, Shevach EM (2007). Autoantigen-specific TGFbeta-induced Foxp3+ regulatory T cells prevent autoimmunity by inhibiting dendritic cells from activating autoreactive T cells. J. Immunol.

[4] Tu E, Bourges D, Gleeson PA, Ang DK, van Driel IR (2013). Pathogenic T cells persist after reversal of autoimmune disease by immunosuppression with regulatory T cells. Eur. J. Immunol.

[5] Xu L, Kitani A, Fuss I, Strober W (2007). Cutting edge: regulatory T cells induce CD4+CD25-Foxp3-T cells or are self-induced to become Th17 cells in the absence of exogenous TGF-beta. J. Immunol.

[6] Zheng SG, Wang J, Horwitz DA (2008). Cutting edge: Foxp3+CD4+CD25+ regulatory T cells induced by IL-2 and TGF-beta are resistant to Th17 conversion by IL-6. J. Immunol.

[7] Yang XO, Nurieva R, Martinez GJ, Kang HS, Chung Y, Pappu BP, Shah B (2008). Molecular antagonism and plasticity of regulatory and inflammatory T cell programs. Immunity.

[8] Stroopinsky D, Avivi I, Rowe JM, Avigan D, Katz T (2009). Allogeneic induced human FOXP3(+)IFN-gamma(+) T cells exhibit selective suppressive capacity. Eur. J. Immunol.

[9] McClymont SA, Putnam AL, Lee MR, Esensten JH, Liu W, Hulme MA, Hoffmuller U (2011). Plasticity of human regulatory T cells in healthy subjects and patients with type 1 diabetes. J. Immunol.

[10] Voo KS, Wang YH, Santori FR, Boggiano C, Wang YH, Arima K, Bover L (2009). Identification of IL-17-producing FOXP3+ regulatory T cells in humans. Proc. Natl. Acad. Sci. USA.

[11] Koenen HJ, Smeets RL, Vink PM, van Rijssen E, Boots AM, Joosten I (2008). Human CD25highFoxp3pos regulatory T cells differentiate into IL-17-producing cells. Blood.

[12] Deknuydt F, Bioley G, Valmori D, Ayyoub M (2009). IL-1beta and IL-2 convert human Treg into T(H)17 cells. Clin.Immunol.

[13] Zhou X, Bailey-Bucktrout SL, Jeker LT, Penaranda C, Martinez-Llordella M, Ashby M, Nakayama M (2009). Instability of the transcription factor Foxp3 leads to the generation of pathogenic memory T cells in vivo. Nat. Immunol.

[14] Bailey-Bucktrout SL, Martinez-Llordella M, Zhou X, Anthony B, Rosenthal W, Luche H, Fehling HJ (2013). Self-antigen-driven activation induces instability of regulatory T cells during an inflammatory autoimmune response. Immunity.

[15] Sakaguchi S, Vignali DA, Rudensky AY, Niec RE, Waldmann H (2013). The plasticity and stability of regulatory T cells. Nat. Rev. Immunol.

[16] O'Connor RA, Leech MD, Suffner J, Hammerling GJ, Anderton SM (2010). Myelin-reactive, TGF-beta-induced regulatory T cells can be programmed to develop Th1-like effector function but remain less proinflammatory than myelin-reactive Th1 effectors and can suppress pathogenic T cell clonal expansion in vivo. J. Immunol.

[17] Ferber IA, Brocke S, Taylor-Edwards C, Ridgway W, Dinisco C, Steinman L, Dalton D (1996). Mice with a disrupted IFN-gamma gene are susceptible to the induction of experimental autoimmune encephalomyelitis (EAE). J. Immunol.

[18] Willenborg DO, Fordham S, Bernard CC, Cowden WB, Ramshaw IA (1996). IFN-gamma plays a critical down-regulatory role in the induction and effector phase of myelin oligodendrocyte glycoprotein-induced autoimmune encephalomyelitis. J. Immunol.

[19] O'Connor RA, Cambrook H, Huettner K, Anderton SM (2013). T-bet is essential for Th1-mediated, but not Th17-mediated, CNS autoimmune disease. Eur. J. Immunol.

[20] Codarri L, Gyulveszi G, Tosevski V, Hesske L, Fontana A, Magnenat L, Suter T (2011). RORgammat drives production of the cytokine GM-CSF in helper T cells, which is essential for the effector phase of autoimmune neuroinflammation. Nat. Immunol.

[21] O'Connor RA, Prendergast CT, Sabatos CA, Lau CW, Leech MD, Wraith DC, Anderton SM (2008). Cutting edge: Th1 cells facilitate the entry of Th17 cells to the central nervous system during experimental autoimmune encephalomyelitis. J. Immunol.

[22] Ponomarev ED, Shriver LP, Maresz K, Pedras-Vasconcelos J, Verthelyi D, Dittel BN (2007). GM-CSF production by autoreactive T cells is required for the activation of microglial cells and the onset of experimental autoimmune encephalomyelitis. J. Immunol.

[23] El-Behi M, Ciric B, Dai H, Yan Y, Cullimore M, Safavi F, Zhang GX (2011). The encephalitogenicity of T(H)17 cells is dependent on IL-1- and IL-23-induced production of the cytokine GM-CSF. Nat. Immunol.

[24] Young A, Linehan E, Hams E, O'Hara Hall AC, McClurg A, Johnston JA, Hunter CA (2012). Cutting edge: suppression of GM-CSF expression in murine and human T cells by IL-27. J. Immunol.

[25] Feng T, Cao AT, Weaver CT, Elson CO, Cong Y (2011). Interleukin-12 converts Foxp3+ regulatory T cells to interferon-gamma-producing Foxp3+ T cells that inhibit colitis. Gastroenterology.

[26] Sawitzki B, Kingsley CI, Oliveira V, Karim M, Herber M, Wood KJ (2005). IFN-gamma production by alloantigen-reactive regulatory T cells is important for their regulatory function in vivo. J. Exp. Med.

[27] Daniel V, Sadeghi M, Wang H, Opelz G (2012). In-vitro inhibition of IFNgamma+ iTreg mediated by monoclonal antibodies against cell surface determinants essential for iTreg function. BMC Immunol.

[28] McGeachy MJ, Stephens LA, Anderton SM (2005). Natural recovery and protection from autoimmune encephalomyelitis: contribution of CD4+CD25+ regulatory cells within the central nervous system. J. Immunol.

[29] O'Connor RA, Malpass KH, Anderton SM (2007). The inflamed central nervous system drives the activation and rapid proliferation of Foxp3+ regulatory T cells. J. Immunol.

[30] O'Connor RA, Floess S, Huehn J, Jones SA, Anderton SM (2012). Foxp3 (+) Treg cells in the inflamed CNS are insensitive to IL-6-driven IL-17 production. Eur. J. Immunol.

[31] Lees JR, Golumbek PT, Sim J, Dorsey D, Russell JH (2008). Regional CNS responses to IFN-gamma determine lesion localization patterns during EAE pathogenesis. J. Exp. Med.

[32] Chen X, Subleski JJ, Kopf H, Howard OM, Mannel DN, Oppenheim JJ (2008). Cutting edge: expression of TNFR2 defines a maximally suppressive subset of mouse CD4+CD25+FoxP3+ T regulatory cells: applicability to tumor-infiltrating T regulatory cells. J. Immunol.

[33] Housley WJ, Adams CO, Nichols FC, Puddington L, Lingenheld EG, Zhu L, Rajan TV (2011). Natural but not inducible regulatory T cells require TNF-alpha signaling for in vivo function. J. Immunol.

[34] Grinberg-Bleyer Y, Saadoun D, Baeyens A, Billiard F, Goldstein JD, Gregoire S, Martin GH (2010). Pathogenic T cells have a paradoxical protective effect in murine autoimmune diabetes by boosting Tregs. J. Clin. Invest.

[35] McPherson RC, Anderton SM (2013). Adaptive immune responses in CNS autoimmune disease: mechanisms and therapeutic opportunities. J. NeuroimmunePharmacol.

[36] Gorelik L, Constant S, Flavell RA (2002). Mechanism of transforming growth factor beta-induced inhibition of T helper type 1 differentiation. J. Exp. Med.

[37] Ince MN, Elliott DE, Setiawan T, Metwali A, Blum A, Chen HL, Urban JF (2009). Role of T cell TGF-beta signaling in intestinal cytokine responses and helminthic immune modulation. Eur. J. Immunol.

[38] Okuda Y, Sakoda S, Bernard CC, Fujimura H, Saeki Y, Kishimoto T, Yanagihara T (1998). IL-6-deficient mice are resistant to the induction of experimental autoimmune encephalomyelitis provoked by myelin oligodendrocyte glycoprotein. Int. Immunol.

[39] Samoilova EB, Horton JL, Hilliard B, Liu TS, Chen Y (1998). IL-6-deficient mice are resistant to experimental autoimmune encephalomyelitis: roles of IL-6 in the activation and differentiation of autoreactive T cells. J. Immunol.

[40] Leech MD, Barr TA, Turner DG, Brown S, O'Connor RA, Gray D, Mellanby RJ (2013). Cutting edge: IL-6-dependent autoimmune disease: dendritic cells as a sufficient, but transient, source. J. Immunol.

[41] Serada S, Fujimoto M, Mihara M, Koike N, Ohsugi Y, Nomura S, Yoshida H (2008). IL-6 blockade inhibits the induction of myelin antigen-specific Th17 cells and Th1 cells in experimental autoimmune encephalomyelitis. Proc. Natl. Acad. Sci. USA.

[42] Suffner J, Hochweller K, Kuhnle MC, Li X, Kroczek RA, Garbi N, Hammerling GJ (2010). Dendritic cells support homeostatic expansion of Foxp3+ regulatory T cells in Foxp3.LuciDTR mice. J. Immunol.

