# Supplementary material for: Exposure to inflammatory cytokines selectively limits GM-CSF production by induced T regulatory cells
Source: Eur J Immunol. 2014 Oct 1;44(11):3342–52. doi: 10.1002/eji.201444687 (PMC4257504; doi:10.1002/eji.201444687)

European Journal of Immunology

Supporting Information

for

DOI 10.1002/eji.201444687

Ben C. Reynolds, Darryl G. Turner, Rhoanne C. McPherson, Catriona T. Prendergast, Richard G. Phelps, Neil A. Turner, Richard A. O'Connor and Stephen M. Anderton

Exposure to inflammatory cytokines selectively limits GM-CSF production by induced T regulatory cells

© 2014 The Authors. *European Journal of Immunology* published by WILEY-VCH Verlag GmbH & Co. KGaA, Weinheim

Supporting Information Fig. 1

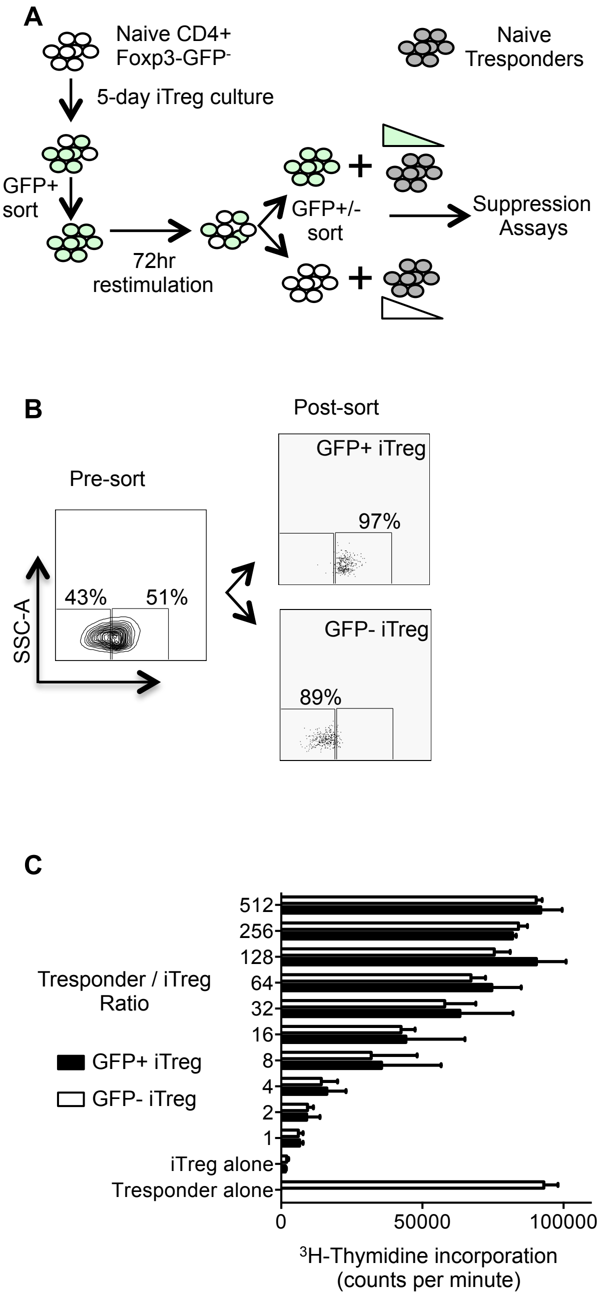

Supporting Information Fig. 2

Gating Strategy for Fig. 4 H-J

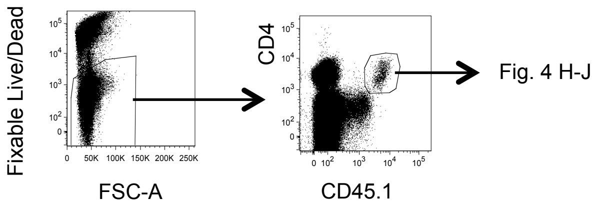

Supporting Information Fig.3

Gating Strategy for Fig. 5 A-G

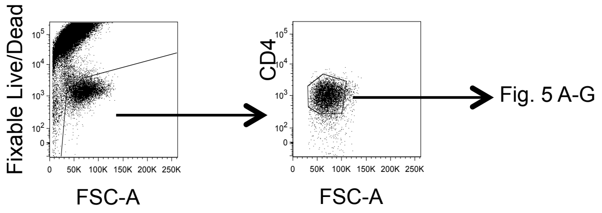

Supplement: Supplementary file 1 [file eji0044-3342-SD1.pdf]
