# Supplementary material for: Exposure to inflammatory cytokines selectively limits GM-CSF production by induced T regulatory cells
Source: Eur J Immunol. 2014 Oct 1;44(11):3342–52. doi: 10.1002/eji.201444687 (PMC4257504; doi:10.1002/eji.201444687)
Supplement: Supplementary file 2 [file eji0044-3342-SD2.pdf]

Peer review correspondence

European Journal of Immunology

Exposure to inflammatory cytokines selectively limits GM-CSF production by induced T regulatory cells

Ben C. Reynolds, Darryl G. Turner, Rhoanne C. McPherson, Catriona T. Prendergast, Richard G. Phelps, Neil A. Turner, Richard A. O'Connor and Stephen M. Anderton

Corresponding author: Stephen M. Anderton, MRC Centre for Inflammation Research, The University of Edinburgh, Queen's Medical Research Institute, Edinburgh, United Kingdom.

Review Timeline:	Submission date:	24 March 2014
	Editorial decision:	29 April 2014
	Revision received:	10 July 2014
	Accepted:	26 August 2014

Handling Executive Committee member: Prof. Shimon Sakaguchi

Please note that the correspondence below does not include the standard editorial instructions regarding preparation and submission of revised manuscripts, only the scientific revisions requested and addressed.

First Editorial Decision – 29 April 2014

Dear Prof. Anderton,

Manuscript ID eji.201444687 entitled "Exposure to inflammatory cytokines selectively limits GM-CSF production by induced T regulatory cells" which you submitted to the European Journal of Immunology has been reviewed. The comments of the referees are included at the bottom of this letter.

A revised version of your manuscript that takes into account the comments of the referees will be reconsidered for publication.

You should also pay close attention to the editorial comments included below. In particular, please edit your figure legends to follow Journal standards as outlined in the editorial comments. Failure to do this will result in delays in the re-review process.

Please note that submitting a revision of your manuscript does not guarantee eventual acceptance, and that your revision will be re-reviewed by the referees before a decision is rendered.

European Journal of Immunology

If the revision of the paper is expected to take more than three months, please inform the editorial office. Revisions taking longer than six months may be assessed by new referee(s) to ensure the relevance and timeliness of the data.

Once again, thank you for submitting your manuscript to European Journal of Immunology and we look forward to receiving your revision.

Yours sincerely,
Laura Soto Vazquez

On behalf of Prof. Shimon Sakaguchi

Editorial Office
European Journal of Immunology
e-mail: ejied@wiley.com
www.eji-journal.eu

****

Reviewer: 1

Comments to the Author
Stability and in vivo efficacy of iTregs is a unresolved issue and most in the field believe that iTregs are not committed Tregs. This work argues that iTregs can be efficacious in vivo in autoimmune settings without stably maintain Foxp3 expression. This is an important message if the authors can substantiate on what makes the cells so effective. Disease inhibition by the iTregs in Figure 4 is striking and perplexing at the same time. It seems this cannot be simply due to lack of GM-CSF production by ex-iTregs at the site of inflammation since GM-CSF production by pathogenic T cells was not changed in the presence of iTregs (Figure 4F). Was clonal expansion of the conventional pathogenic Tg4 cells inhibited by the iTregs? Did the iTregs expand themselves after in vivo transfer? Given the essential role of IL-17 in EAE, the authors should look at IL-17 production by the (ex)-iTreg and pathogenic cells in vivo.

This manuscript provides useful information on the characteristics and behavior of in vitro induced Tregs. It would be very helpful if the authors have provided information on naive cells cultured under neutral condition so comparison between iTregs, ex-iTregs, and conventional T cells can be made. For example, the selective inhibition of GM-CSF in ex-iTregs by IL-27, IL-12 and IL-6 is very interesting (Figure 5). Is this also true for conventional T cells or specific for (ex)-iTregs? It has been argued that IFNg production

European Journal of Immunology

by Tregs can be essential for their suppressive function in vivo (Dan Campbell and Kathryn Wood) and what distinguishes the suppressive IFNg from Tregs and pathogenic IFNg by conventional cells is the amount that these two cell types produce. Providing data on control conventional T cell will make it easier for readers to appreciate the magnitude of cytokine production by iTregs in comparison with pathogenic conventional T cells.

Reviewer: 2

Comments to the Author
This is a strong manuscript in an important research area that has clear translational relevance. The study demonstrates that induced Treg retain the capacity to suppress in vivo (as assessed by inhibition of EAE) despite significant production of pro-inflammatory cytokines. In addition, despite the common perception that an inflammatory environment will provoke cytokine production from Treg, the authors actually demonstrate that trans-differentiated Treg produce less GM-CSF in the EAE setting and that this likely reflects the actions of cytokines such as IL-27, IL-12 and IL-6. The capacity of the inflammatory environment to selectively shape cytokine output from Treg populations (e.g. maintaining IFNg yet decreasing GM-CSF) is interesting and novel.

One point was a little unclear to me in the discussion – the authors state that the production of pro-inflammatory cytokines by iTreg “does not appear to represent trans-differentiation of these cells” i.e. the loss of Foxp3. However my take on the presented data is that Treg can produce small amounts of cytokine without losing Foxp3 (Fig 2) but that when restimulated either in vitro (Fig 1) or in vivo (Fig 4) the cytokines that are produced mainly derive from trans-differentiated (Foxp3-negative) cells.

An additional point for clarity – when the authors state that “irradiated splenic APC” were used in the in vitro assays, does this simply mean red-cell-lysed spleen cells or were the splenocytes T-depleted or lymphocyte-depleted?

Reviewer: 3

Comments to the Author
The paper by Reynolds, et al. entitled ‘Exposure to inflammatory cytokines selectively limits GM-CSF production by induced T regulatory cells’ describes a role of in vitro-generated iTreg expressing inflammatory cytokines in the suppression of effector T cells that cause neuroinflammation, EAE. This paper represents a potential usefulness of iTreg-based therapy in the future even though iTreg produce inflammatory cytokines such as IFNg, TNF, and GM-CSF and lose Foxp3 expression. Following the

European Journal of Immunology

transfer of iTreg, an environmental cue in the inflamed tissue and the dynamics in cytokine receptors on iTregs confer the suppressive function in iTregs. These findings are intriguing and original, but a few questions remain to be answered in order to improve the paper since the novelty of the paper is weak and limited.

Major concerns;

1. As the authors are aware of the fact “Loss of Foxp3 expression by iTreg is widely described” on Page4, in vitro-generated iTreg are not a stable Foxp3+ subset and the vast majority of them loses their Foxp3 expression in vitro and in vivo after transfer as shown in the figures even after FACS purified on the basis of Foxp3-GFP expression. In accordance with the cellular instability, those iTregs generally show imcomplete demethylation of the Treg-specific demethylation region (TSDR) on the Foxp3 locus. As shown in Figure 4, Tg4 iTreg with MBP-specific TCR maintained Foxp3 expression only in 10% of transferred cells, but completely protected mice from EAE, suggesting that truly converted iTreg (10%) in vivo may have good suppression function over effector T cells or exFoxp3 cells (90%), which were Foxp3+ once and have lost Foxp3, may acquire suppressive capacity independent of Foxp3. One of my major questions is whether remaining 10% Foxp3+ cells are important or exFoxp3-positive cells, which are producing inflammatory cytokines, are still somehow suppressive. Since the transfer of MBP-specific iTreg alone did not cause EAE, the second transfer experiment of exFoxp3 cells from these EAE protected mice would be a key experiment to fully support the author’s idea that a therapy using iTreg, which have once become Foxp3+ in vitro, would be safe even if acquiring an inflammatory profile with self-antigen specificities following transfer.

2. Relating to the above, the authors also can test iTreg suppression function using exFoxp3 cells shown in Figure5. 70-90% of GFP+ Foxp3+ cells lost Foxp3 expression 3 days after IL-1, IL-6, IL-12, and IL-27 treatment in vitro. It would be worth testing whether exFoxp3 (GFP-negative) cells from Tg4 mice are pathogenic or suppressive.

First revision – authors’ response – 10 July 2014

Reviewer: 1

Comments to the Author

Stability and in vivo efficacy of iTregs is a unresolved issue and most in the field believe that iTregs are not committed Tregs. This work argues that iTregs can be efficacious in vivo in autoimmune settings without stably maintain Foxp3 expression. This is an important message if the authors can substantiate on what makes the cells so effective.

European Journal of Immunology

Disease inhibition by the iTregs in Figure 4 is striking and perplexing at the same time. It seems this cannot be simply due to lack of GM-CSF production by ex-iTregs at the site of inflammation since GM-CSF production by pathogenic T cells was not changed in the presence of iTregs (Figure 4F).
Was clonal expansion of the conventional pathogenic Tg4 cells inhibited by the iTregs?

Response:
Clonal expansion of conventional cells was indeed inhibited by iTreg. These data are shown in Fig. 4C&D. Although the percentage of GM-CSF+ conventional Tg4 cells was not reduced in the presence of iTreg, the greatly reduced clonal expansion means that the absolute numbers of GM-CSF+ cells is similarly low. This effect was described in the original text (page 5, last paragraph).

Comment:
Did the iTregs expand themselves after in vivo transfer?

Response:
This is difficult to determine definitively. The numbers of iTreg that could be identified in the lymph nodes seven days after immunization were very much lower than the 2x106 that we transferred. Looking earlier, at 2 days after immunization, showed that inclusion of antigen elevated the numbers and frequencies of iTreg within the draining lymph node, but we could not discern whether this was a result of their improved localization at the site of antigen presentation and/or proliferation. Definitive information on this requires further studies. The data shown in Fig. 4H were from 7 days post-immunization using spleen, in which sufficient iTreg could be identified for cytokine analysis.

Comment:
Given the essential role of IL-17 in EAE, the authors should look at IL-17 production by the (ex)-iTreg and pathogenic cells in vivo.

Response:
There are several reasons for not investigating IL-17 in our iTreg.
a) Whether CD4+ T cell-derived IL-17 is essential for the development of EAE is in fact far from certain (e.g. Haak, et al JCI, 119:61; 2009)
b) GM-CSF is now considered to be the key T cell-derived cytokine, needed for EAE development (Codarri, et al, Nat Immunol., 12:560; 2011).
c) Most importantly, our previous publication (O'Connor et al., JI, 185: 7235; 2010; cited as reference 16) described that iTreg do not in fact produce IL-17 in response to secondary stimulation, even when exposed to a standard "Th17" cytokine cocktail. This is because of their early production of IFN-γ and expression of T-bet during the initial Foxp3-inducing culture. Thus we do not show IL-17 production, because none can be measured from iTreg.

European Journal of Immunology

We describe this last point in the introduction, as part of our rationale for the current study.

Comment:
This manuscript provides useful information on the characteristics and behavior of in vitro induced Tregs. It would be very helpful if the authors have provided information on naive cells cultured under neutral condition so comparison between iTregs, ex-iTregs, and conventional T cells can be made. For example, the selective inhibition of GM-CSF in ex-iTregs by IL-27, IL-12 and IL-6 is very interesting (Figure 5). Is this also true for conventional T cells or specific for (ex)-iTregs? It has been argued that IFNg production by Tregs can be essential for their suppressive function in vivo (Dan Campbell and Kathryn Wood) and what distinguishes the suppressive IFNg from Tregs and pathogenic IFNg by conventional cells is the amount that these two cell types produce. Providing data on control conventional T cell will make it easier for readers to appreciate the magnitude of cytokine production by iTregs in comparison with pathogenic conventional T cells.

Response:
We have now compared the effects of IL-6, IL-12 and IL-27 on cytokine production by iTreg versus Th0 and Th1 cells. The results indicate differential effects on each cell type which we have detailed in the manuscript with an additional Fig. 6, and a corresponding new section in the Results and some additional text in the Discussion.

Reviewer: 2

Comments to the Author
This is a strong manuscript in an important research area that has clear translational relevance. The study demonstrates that induced Treg retain the capacity to suppress in vivo (as assessed by inhibition of EAE) despite significant production of pro-inflammatory cytokines. In addition, despite the common perception that an inflammatory environment will provoke cytokine production from Treg, the authors actually demonstrate that trans-differentiated Treg produce less GM-CSF in the EAE setting and that this likely reflects the actions of cytokines such as IL-27, IL-12 and IL-6. The capacity of the inflammatory environment to selectively shape cytokine output from Treg populations (e.g. maintaining IFNg yet decreasing GM-CSF) is interesting and novel.

One point was a little unclear to me in the discussion – the authors state that the production of pro-inflammatory cytokines by iTreg “does not appear to represent trans-differentiation of these cells” i.e. the loss of Foxp3. However my take on the presented data is that Treg can produce small amounts of

European Journal of Immunology

cytokine without losing Foxp3 (Fig 2) but that when restimulated either in vitro (Fig 1) or in vivo (Fig 4) the cytokines that are produced mainly derive from trans-differentiated (Foxp3-negative) cells.

Response:
We agree that this phrase was rather ambiguous in the original manuscript. We were trying to convey that trans-differentiation was not occurring during the initial culture. We have decided that the simplest remedy is to delete this sentence form the text.

Comment:
An additional point for clarity – when the authors state that “irradiated splenic APC” were used in the in vitro assays, does this simply mean red-cell-lysed spleen cells or were the splenocytes T-depleted or lymphocyte-depleted?

Response:
These were red cell-lysed B10.PL or C57BL/6xB10.PL splenocytes.
We have clarified this in the Materials & Methods.

Reviewer: 3

Comments to the Author
The paper by Reynolds, et al. entitled ‘Exposure to inflammatory cytokines selectively limits GM-CSF production by induced T regulatory cells’ describes a role of in vitro-generated iTreg expressing inflammatory cytokines in the suppression of effector T cells that cause neuroinflammation, EAE. This paper represents a potential usefulness of iTreg-based therapy in the future even though iTreg produce inflammatory cytokines such as IFNg, TNF, and GM-CSF and lose Foxp3 expression. Following the transfer of iTreg, an environmental cue in the inflamed tissue and the dynamics in cytokine receptors on iTregs confer the suppressive function in iTregs. These findings are intriguing and original, but a few questions remain to be answered in order to improve the paper since the novelty of the paper is weak and limited.

Major concerns;
1. As the authors are aware of the fact “Loss of Foxp3 expression by iTreg is widely described” on Page4, in vitro-generated iTreg are not a stable Foxp3+ subset and the vast majority of them loses their Foxp3 expression in vitro and in vivo after transfer as shown in the figures even after FACS purified on the basis of Foxp3-GFP expression. In accordance with the cellular instability, those iTregs generally show imcomplete demethylation of the Treg-specific demethylation region (TSDR) on the Foxp3 locus. As shown in Figure 4, Tg4 iTreg with MBP-specific TCR maintained Foxp3 expression only in 10% of

European Journal of Immunology

transferred cells, but completely protected mice from EAE, suggesting that truly converted iTreg (10%) in vivo may have good suppression function over effector T cells or exFoxp3 cells (90%), which were Foxp3+ once and have lost Foxp3, may acquire suppressive capacity independent of Foxp3. One of my major questions is whether remaining 10% Foxp3+ cells are important or exFoxp3-positive cells, which are producing inflammatory cytokines, are still somehow suppressive. Since the transfer of MBP-specific iTreg alone did not cause EAE, the second transfer experiment of exFoxp3 cells from these EAE protected mice would be a key experiment to fully support the author's idea that a therapy using iTreg, which have once become Foxp3+ in vitro, would be safe even if acquiring an inflammatory profile with self-antigen specificities following transfer.

Response:
These are of course interesting questions that we have considered carefully. Our experience with these models tells us that the idea of transferring iTreg, allowing some to lose Foxp3 in vivo and re-isolating sufficient Foxp3- cells, for functional comparison with their (fewer) Foxp3+ counterparts by secondary transfer would be beyond our capabilities in terms of the large numbers starting TCR tg cell populations and primary host mice that would be required.

2. Relating to the above, the authors also can test iTreg suppression function using exFoxp3 cells shown in Figure5. 70-90% of GFP+ Foxp3+ cells lost Foxp3 expression 3 days after IL-1, IL-6, IL-12, and IL-27 treatment in vitro. It would be worth testing whether exFoxp3 (GFP-negative) cells from Tg4 mice are pathogenic or suppressive.

Response:
iTreg are quite fragile following secondary stimulation and collecting sufficient viable cells by FACS sorting for in vivo transfer would be very demanding. However, we have been able recover sufficient cells to test the relative suppressive qualities of GFP+ versus GFP- cells following secondary stimulation, using in vitro suppression assays. We have included these data as Supporting Information Fig. 1. These data show essentially identical suppressive efficacy for each population across a range of cell numbers. Although the purity of GFP- cells was consistently not as high as desired (~ 90% GFP-), we would expect suppression to titrate out rapidly if it was due to contaminating GFP+ cells. We did not see this. This suggests that "ex-iTreg" retain suppressive activity (at least in vitro) for a period once Foxp3-expression is lost. We have added a section to the Results to address this. We would stress that a full understanding of basis for this remains beyond the scope this current paper.

We should also emphasise that we are not attempting to assert that the loss of FoxP3 from iTreg population has any therapeutic advantage.

Peer review correspondence

European Journal of Immunology

Second Editorial Decision – 14 August 2014

Dear Prof. Anderton,

It is a pleasure to provisionally accept your manuscript entitled "Exposure to inflammatory cytokines selectively limits GM-CSF production by
induced T regulatory cells" for publication in the European Journal of Immunology. For final acceptance, please follow the instructions below and return the requested items as soon as possible as we cannot process your manuscript further until all items listed below are dealt with.

Please note that EJI articles are now published online a few days after final acceptance (see Accepted Articles: http://onlinelibrary.wiley.com/journal/10.1002/(ISSN)1521-4141/accepted). The files used for the Accepted Articles are the final files and information supplied by you in Manuscript Central. You should therefore check that all the information (including author names) is correct as changes will NOT be permitted until the proofs stage.

We look forward to hearing from you and thank you for submitting your manuscript to the European Journal of Immunology.

Yours sincerely,
Karen Chu

on behalf of Prof. Shimon Sakaguchi

Dr. Karen Chu
Editorial Office
European Journal of Immunology
e-mail: ejied@wiley.com
www.eji-journal.eu
